# Argininosuccinate lyase is a metabolic vulnerability in breast development and cancer

**DOI:** 10.1038/s41540-021-00195-5

**Published:** 2021-09-17

**Authors:** Sigurdur Trausti Karvelsson, Qiong Wang, Bylgja Hilmarsdottir, Arnar Sigurdsson, Siver Andreas Moestue, Gunhild Mari Mælandsmo, Skarphedinn Halldorsson, Steinn Gudmundsson, Ottar Rolfsson

**Affiliations:** 1 Center for Systems Biology, University of Iceland, Reykjavik, Iceland; 2 Department of Tumor Biology, Institute for Cancer Research, Oslo University Hospital, Oslo, Norway; 3 Department of Chemistry, Sekr. TC 2, Faculty II Mathematics and Natural Sciences, Technische Universität Berlin, Berlin, Germany; 4 Department of Clinical and Molecular Medicine, NTNU, Trondheim, Norway; 5 Department of Pharmacy, Nord University, Namsos, Norway; 6 Institute for Surgical Research, Vilhelm Magnus Laboratory, Oslo University Hospital, Oslo, Norway; 7 School of Engineering and Natural Sciences, University of Iceland, Reykjavik, Iceland

**Keywords:** Biomarkers, Biochemical networks, Stem cells, Computer modelling, Cancer

## Abstract

Epithelial-to-mesenchymal transition (EMT) is fundamental to both normal tissue development and cancer progression. We hypothesized that EMT plasticity defines a range of metabolic phenotypes and that individual breast epithelial metabolic phenotypes are likely to fall within this phenotypic landscape. To determine EMT metabolic phenotypes, the metabolism of EMT was described within genome-scale metabolic models (GSMMs) using either transcriptomic or proteomic data from the breast epithelial EMT cell culture model D492. The ability of the different data types to describe breast epithelial metabolism was assessed using constraint-based modeling which was subsequently verified using ^13^C isotope tracer analysis. The application of proteomic data to GSMMs provided relatively higher accuracy in flux predictions compared to the transcriptomic data. Furthermore, the proteomic GSMMs predicted altered cholesterol metabolism and increased dependency on argininosuccinate lyase (ASL) following EMT which were confirmed in vitro using drug assays and siRNA knockdown experiments. The successful verification of the proteomic GSMMs afforded iBreast2886, a breast GSMM that encompasses the metabolic plasticity of EMT as defined by the D492 EMT cell culture model. Analysis of breast tumor proteomic data using iBreast2886 identified vulnerabilities within arginine metabolism that allowed prognostic discrimination of breast cancer patients on a subtype-specific level. Taken together, we demonstrate that the metabolic reconstruction iBreast2886 formalizes the metabolism of breast epithelial cell development and can be utilized as a tool for the functional interpretation of high throughput clinical data.

## Introduction

Roughly 90% of all cancer-related deaths are believed to be caused by secondary metastatic tumors^1^. Multiple enzymes have been identified that support cancer cell dissemination in breast cancer through alterations of core metabolic pathways. These include the glycolytic enzymes HK1 and PKM2^2,3^, IDH1 involved in the tricarboxylic acid (TCA) cycle^4^, ACLY in fatty acid synthesis^5^, and PRODH from proline synthesis^6^. Definitive metabolic patterns that differentiate between invasive and non-invasive cancer cells however remain elusive^7^.

One way that epithelial cells gain invasive properties is through the developmental process known as epithelial-to-mesenchymal transition (EMT). When localized breast cancer epithelial cells go through EMT, they gain invasive and apoptosis-resistant properties that contribute to their ability to migrate through the extracellular matrix and form secondary tumors through mesenchymal-to-epithelial transition (MET)^8–10^. Metabolic alterations are believed to be a hallmark of cancer and tumor progression^11^ and thus, an overall understanding of the metabolic changes that accompany EMT and MET in breast tissue may help to recognize potential biomarkers and drug targets associated with cancer progression.

Genome-scale metabolic models (GSMMs) have been successfully used to analyze and interpret changes to cancer metabolism based upon high-throughput datasets^12–14^. GSMM-based studies have revealed significant alterations in the reducing potential during breast tumor development where NADPH is increasingly directed towards reactive oxygen species (ROS) defenses^15^. Furthermore, the predicted metabolic variability between patients has been utilized successfully for their prognosis^14^. These studies^14,15^ were based on transcriptomic or proteomic data obtained from the cell lines or tumors of interest but lacked direct measurements of uptake/secretion rates that constrain metabolic flux as these measurements are challenging to obtain in a clinical setting. Directly incorporating metabolic measurements is expected to provide more accurate predictions than clinical breast cancer data alone.

We hypothesize that GSMMs representing the metabolic plasticity of EMT may help define the metabolism of breast tissue and contribute to the identification of metabolic vulnerabilities for breast cancer diagnostic or therapeutic purposes. The epithelial-derived D492 cell EMT model is comprised of two cell lines (D492 and D492M) that allow metabolic differences that occur following spontaneous EMT in cell culture to be investigated^16^. Similar cell models previously used to study EMT include HMLE and the PMC42 EMT cell models^17–19^.

In order to describe the metabolic plasticity of EMT we recently reported the metabolism of D492 and its mesenchymal-like counterpart D492M by integrated analyses of extracellular metabolomic- and transcriptomic data within tailored GSMMs. The metabolic alterations that occur following EMT in D492^16^ mirrored results from a comprehensive analysis of EMT metabolism^20^ and anchorage-independent growth^21^. A decrease in glycolysis and changes to mitochondrial oxidation of amino acids, specifically glutamine, threonine, arginine and lysine were observed. Those analyses were limited to transcriptomic and extracellular metabolomics data prompting the question of how proteomic data would alter the predictions of D492 metabolic network activity given the nonlinear relationship of transcription and translation^22,23^.

Here, we extend the D492 EMT GSMM, now termed iBreast2886, to include differences in protein levels, further formulating the metabolism of EMT and investigate the models ability to describe breast tissue metabolism. In order to capture the intracellular metabotypes that accompany EMT in D492 and identify biomarkers that discriminate between the two phenotypes, we used constraint-based modeling and comparative metabolic analysis. In order to reconcilidate the predicted differences in metabolic phenotypes based on the different data types, we carried out enzyme inhibitor assays, 1-^13^C-glutamine tracer analyses, and siRNA knockdown experiments in vitro to determine the actual phenotypes D492 and D492M cells. Finally, we demonstrate how iBreast2886 can be used as a tool for functional interpretation of tumor gene expression data from breast cancer patients.

## Results

### Direct comparison of different data types reveals their low overlap

**Fig. 1:**
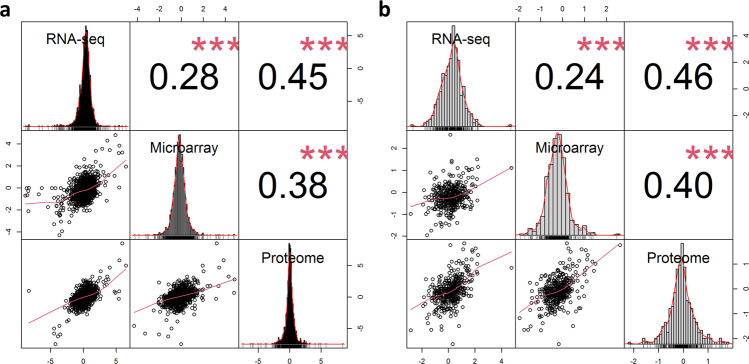
Correlation of the log-fold differences in D492 and D492M of common gene identifiers between RNA sequencing, microarray and proteomic data. **a** Correlation of log-fold differences of all common gene identifiers (*n* = 2271). **b** Correlation of log-fold differences of common metabolic gene identifiers (*n* = 395). Spearman’s rank correlation coefficient was used. The asterisks represent a significant correlation (*p* < 0.001).

In order to determine the consistency of the three different types of data used in this study (microarray, RNA sequencing (RNA-seq) and proteomic) for D492 epithelial cells and D492M mesenchymal cells, we compared the three data types by calculating the Spearman correlation of the log-fold differences between D492M and D492 (Fig. 1a).

The correlation between RNA-seq and proteomic data was the highest (*ρ* = 0.46) and the correlation between the two gene expression methods was lowest (*ρ* = 0.28). By comparing only the metabolic identifiers, the correlation between the dataset did not change (Fig. 1b).

To compare the datasets on a more functional metabolic level, we investigated and compared their ability to infer metabolic activity of D492 and D492M using constraint-based metabolic modeling^24^. In order to achieve this, we used the different datasets as constraints on our previous reconstruction of breast metabolism, which we refer to hereafter as iBreast2886.

### True metabolic flux is reflected in cell-specific metabolic networks from proteomic data but not other data types

For the comparative metabolic analysis, we constructed GSMMs based on RNA-seq and proteomic data from the epithelial D492 and mesenchymal D492M and compared these to microarray-based GSMMs built previously^16^ and iBreast2886 GSMMs where only the extracellular constraints were applied. Henceforth, these will be referred to as the RNA-seq GSMMs, protein GSMMs, microarray GSMMs, and media GSMMs.

**Fig. 2:**
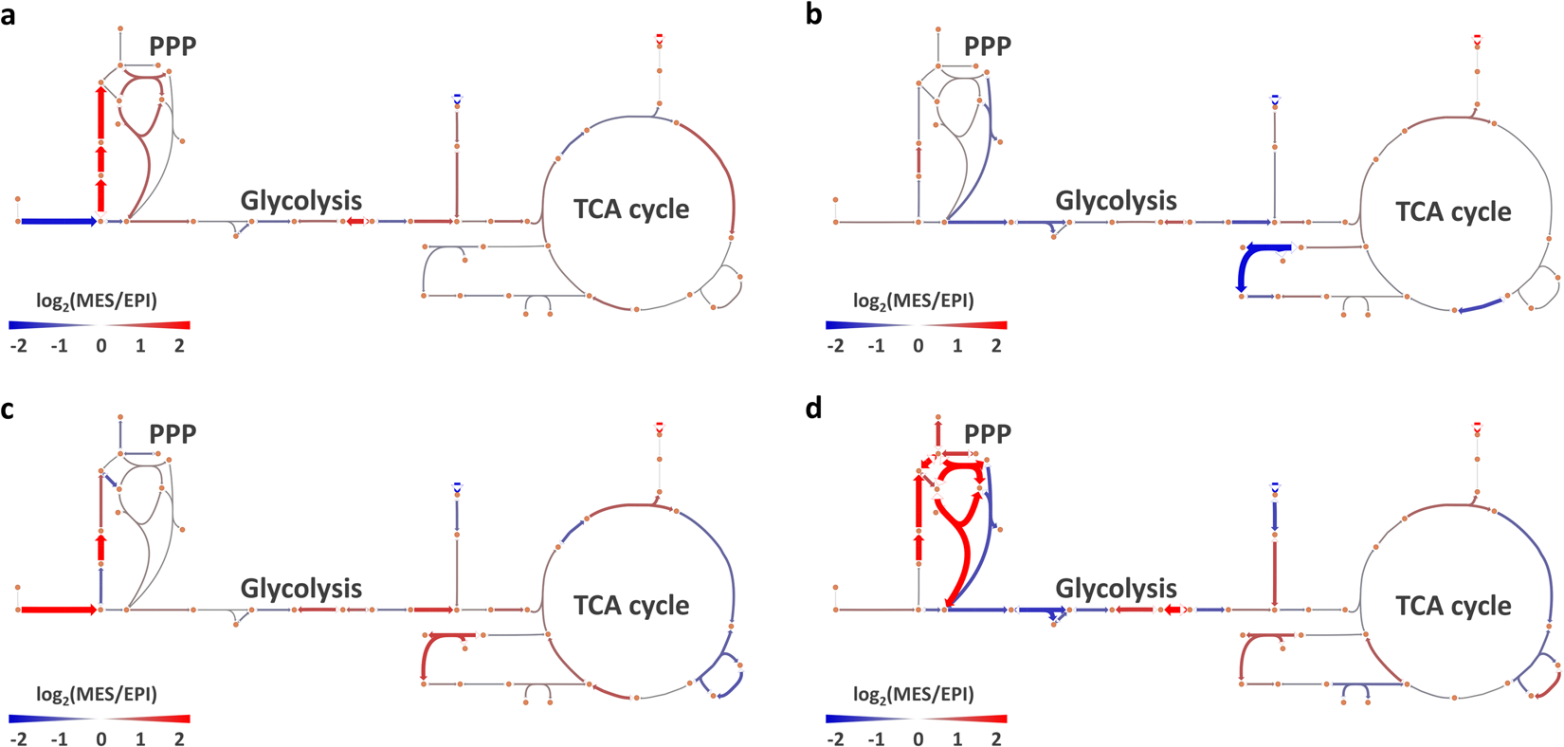
Relative differences in reaction activity in EPI and MES models constrained in four different ways. **a** Only extracellular constraints, (**b**) Microarray, (**c**) RNA-seq and (**d**) Proteomic data. The pathways shown are glycolysis, TCA cycle, and pentose-phosphate pathway. Red represents higher activity in MES whereas blue represents higher activity in EPI, represented by log-fold differences in median activity from random sampling of the models.

In order to compare the EPI and MES models in all pairs of GSMMs, representative flux values (flux profiles) for all reactions that obey the GSMM steady-state assumptions for all models were obtained through random sampling of the solution space^25^, where the median values for reactions were used to represent their activity. The relative differences between EPI and MES in all four GSMM pairs are summarized in Fig. 2a–d. Hierarchical clustering of the GSMMs flux profiles revealed highest similarity between the RNA-seq- and proteomic-constrained models on a phenotype-specific level (Supplementary figure 1). Upon closer inspection, it was clear that reaction similarity was different in the various subcellular compartments. Specifically, the flux similarity of the RNA-seq- and proteomic-constrained models was compartment specific, where the endoplasmic reticulum (ER) had the highest correlation of EMT-linked differences in reaction activity, followed by the cytosol and mitochondria (Supplementary table 1).

**Fig. 3:**
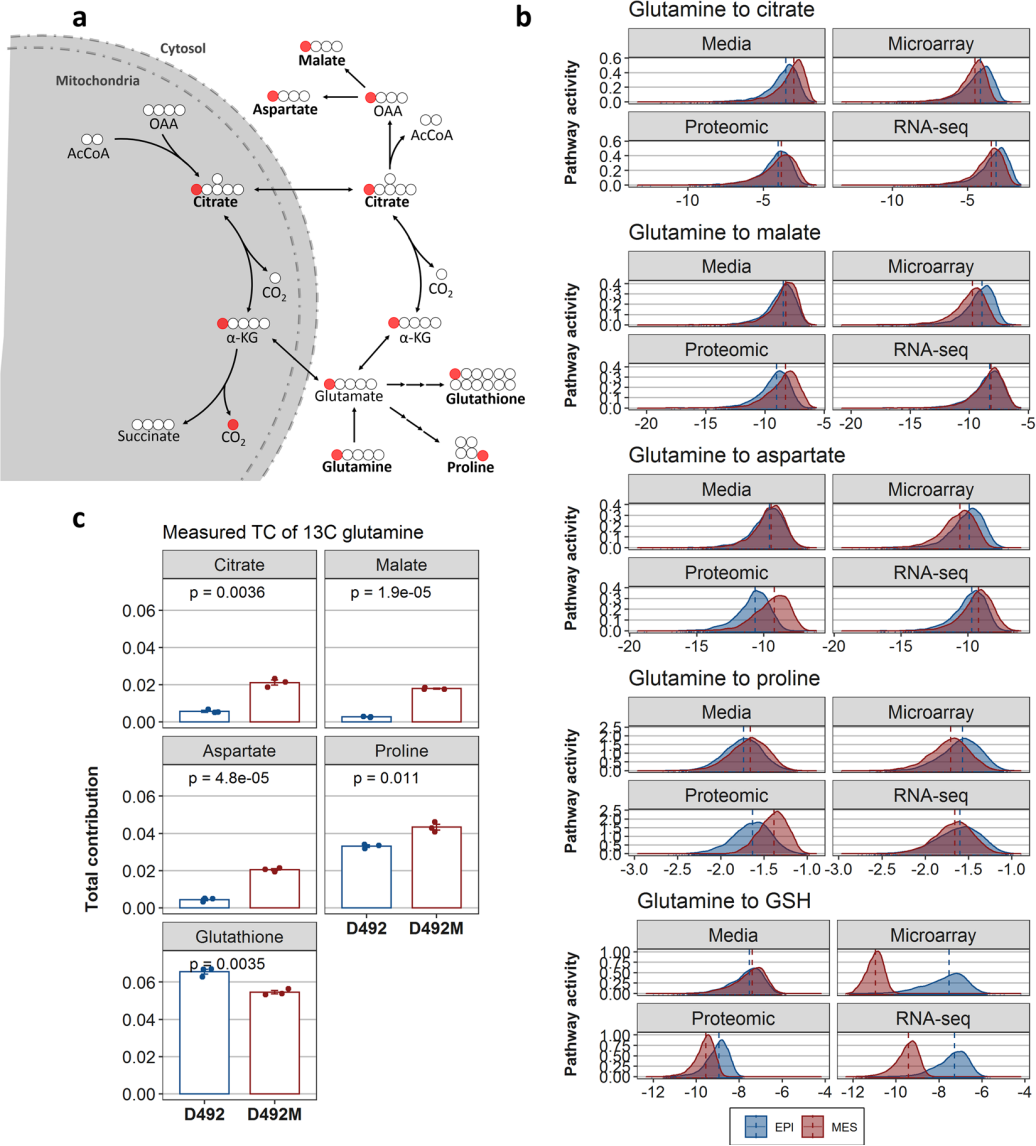
Predicted and measured metabolic route activity in D492 and D492M cells. **a** A carbon tracing map of 1-^13^C-labeled glutamine describing the flow and fate of labeled carbons in the glutamine carbon skeleton. Metabolites in bold are the end metabolites within the pathways we quantified. Red circles represent the ^13^C- carbon isotopes. The mitochondria is indicated by shaded grey. **b** Density plots of the calculated metabolic route activitiy (MRA) of five different routes of reductive glutamine metabolism from the total random sampling matrix (*n* = 5800 flux vectors) for all GSMMs. The blue distributions represent MRA within the epithelial GSMMs whereas red represents MRA within the mesenchymal GSMMs. The dashed line represents the median MRA value. Higher (i.e. more positive) values represent more active routes. All distributions were significantly different in (**b**) (*p* < 0.001) based on a Kolmogorov–Smirnov test. **c** Measured total contribution (TC) from 1-^13^C-glutamine to selected metabolites (after 6 h of cell culture) which represent the same metabolic routes as in (**b**). Results in (**c**) are shown as mean + SEM from three experiments (shown with dots). Student’s *t*-test was used to estimate significance.

As the ground truth for the comparative analysis of pathway activity within our GSMMs, we used isotope labeling patterns from 1-^13^C-labeled glutamine experiments. This tracer is capable of quantifying the contribution of glutamine, one of two major carbon sources of D492 and D492M^16^, to citrate, malate, and aspartate through reductive carboxylation (Fig. 3a) and to the synthesis of proline and glutathione. The contributions from glutamine to the aforementioned metabolites are not whole metabolic pathways but subsets of reductive glutaminolysis and will be referred to as metabolic routes. Some of these metabolic routes occur in more than one cellular compartment. Reductive glutaminolysis is therefore a good representation of the compartment-based complexity of eukaryotic cellular metabolism.

It is challenging to infer metabolic pathway activity by observing multiple, individual reactions (c.f Fig. 2). Therefore, we utilized an activity measure that quantifies metabolic route activity in compartmentalized GSMMs based on random sampling results. From the metabolic route activity calculations, we observed that the different omics-constrained GSMMs had different predictions of the production of metabolites derived from glutamine (Fig. 3b). According to the 1-^13^C-labeled glutamine results, there was relatively higher citrate derived from glutamine in D492M than D492, indicating increased flow through reductive carboxylation of glutamine and/or the decreased condensation of oxaloacetate and acetyl-CoA (Fig. 3a). The proteomic GSMMs were the only ones to predict both routes correctly (Fig. 3c and Supplementary figure 2) along with the other four routes that were investigated. The microarray-constrained GSMMs correctly predicted the relative difference in metabolic route activity for only two routes, the RNA-seq GSMMs for three routes, and the GSMMs constrained only with extracellular uptake and secretion rates predicted correctly for four routes in total. Thus, the results indicated the relatively higher validity of the proteomics-constrained GSMMs compared to the other data types for intracellular, compartmentalized flux predictions.

### Results from GSMMs constrained with proteomic data reveal metabolic vulnerabilities of EMT

**Fig. 4:**
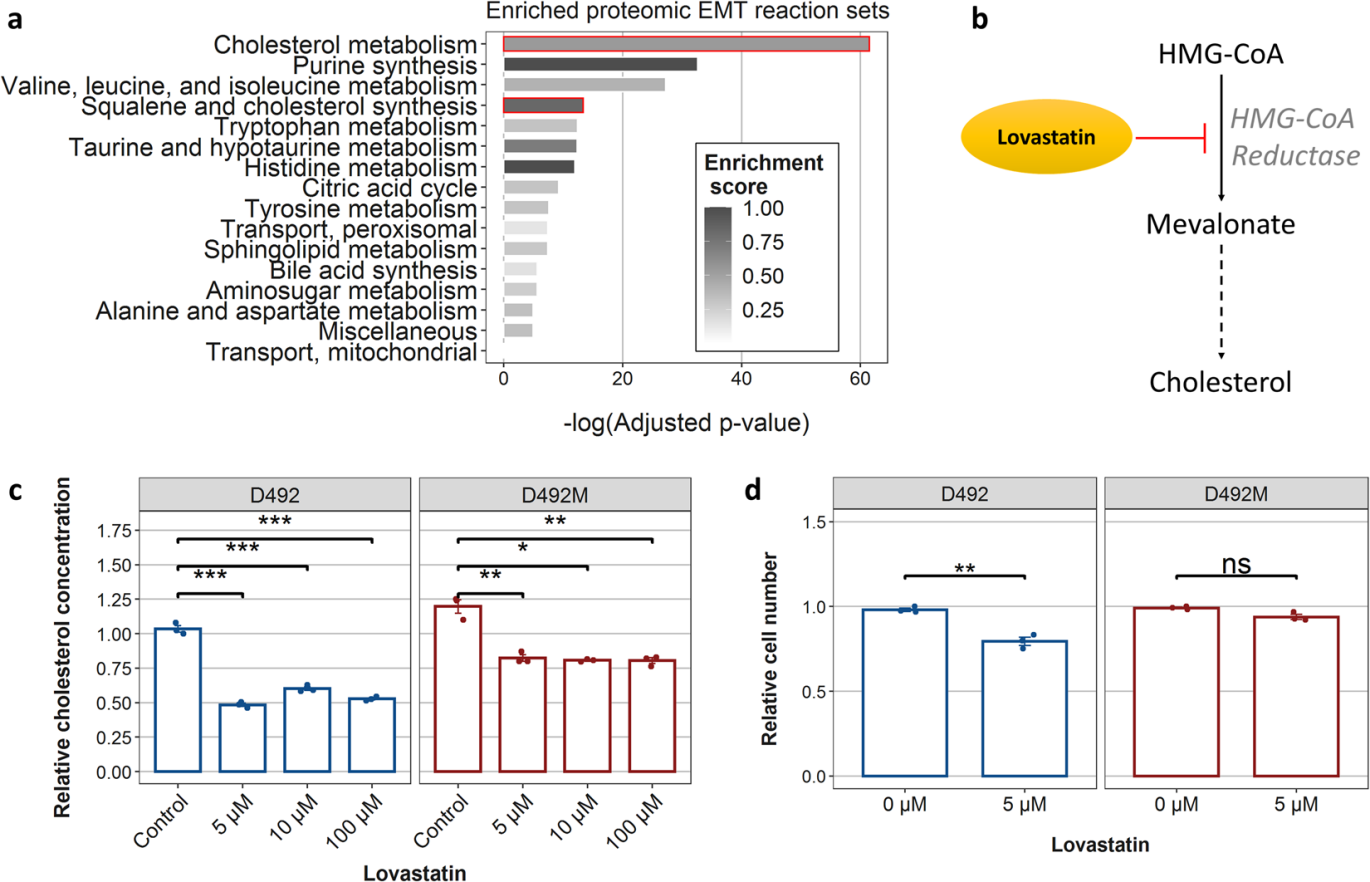
Integrated analysis of all data-type based GSMMs reveals EMT-related differences in cholesterol metabolism. **a** Enrichment analysis of reactions that need alterations for the EPI to take on a MES flux phenotype in all proteomic GSMMs. This approach helps to identify reaction sets (i.e., families of pathways) which need to be altered for EMT-related changes in flux profiles. Reaction sets shown are ones with FDR-corrected *p* value less than 0.01. The scale (Enrichment score) represents the fraction of set reactions within the EMT reactions. The *p* values are FDR-adjusted *p* values from a hypergeometric test for enrichment of reaction sets. The two reaction sets highlighted in red are cholesterol-related. **b** The mechanism of inhibition of cholesterol synthesis by lovastatin. Dashed arrows represent more than one metabolic reaction. **c** Both D492 and D492M were treated with various concentrations of lovastatin, an inhibitor of cholesterol synthesis. The figure shows the cholesterol concentration in D492 and D492M cells after treatment with lovastatin. **d** Survival of D492 and D492M cells after treatment 5 µM concentration of lovastatin. Results in c) and d) are shown as mean + SEM from three experiments (shown with dots). Student’s *t*-test was used to estimate significance and *p* values were adjusted using the Benjamini–Hochberg approach.

For the investigation of EMT-specific metabolic remodeling, we utilized the same methodology as before^16^ to identify reactions whose activity specifically requires alteration in order to switch from a epithelial flux profile (EPI) to a mesenchymal one (MES). As the proteomics-constrained GSMMs had the most accurate flux predictions, we used them for this analysis. Briefly, we used a hypergeometric test to identify whether the altered reactions were enriched with any subsystems (*e.g*. the metabolic pathway families with specific functional roles) within iBreast2886. The results showed that two out of the top four enriched reaction sets among EMT-linked reactions are within cholesterol metabolism (highlighted in red in Fig. 4a).

Statins are a class of drugs that are broadly prescribed to patients with hypercholesterolemia. They work by inhibiting HMG-CoA reductase (Fig. 4b), the rate-limiting step in cholesterol synthesis^26^. We treated the D492 and D492M cells with lovastatin and found that following the successful inhibition of cholesterol synthesis in both cell lines (Fig. 4c), it was apparent that the D492 cells were more sensitive to the drug in terms of survival (Fig. 4d).

Thus, in addition to being the most accurate model in terms of intracellular fluxes, the analysis of the proteomic iBreast2886 GSMMs proved useful in identifying the differences in cholesterol metabolism in D492 and D492M. On the same note, we performed gene essentiality analysis of the proteomic GSMMs and found the essential genes for EPI and MES, respectively. Focusing particularly on the mesenchymal GSMM due to its metastatic involvement, we found that there were nine genes essential for the MES model. These were Argininosuccinate Lyase (*ASL)*, Ornithine Aminotransferase *(OAT)*, Pyruvate Dehydrogenase Complex Component X *(PDHX)*, Proline Dehydrogenase 1 *(PRODH)*, Renin binding protein *(RENBP)*, Isocitrate Dehydrogenase 2 (*IDH2*), Guanylate Kinase 1 (*GUK1*), 6-Phosphogluconolactonase (*PGLS*) and Cystathionine Gamma-Lyase (*CTH*).

**Fig. 5:**
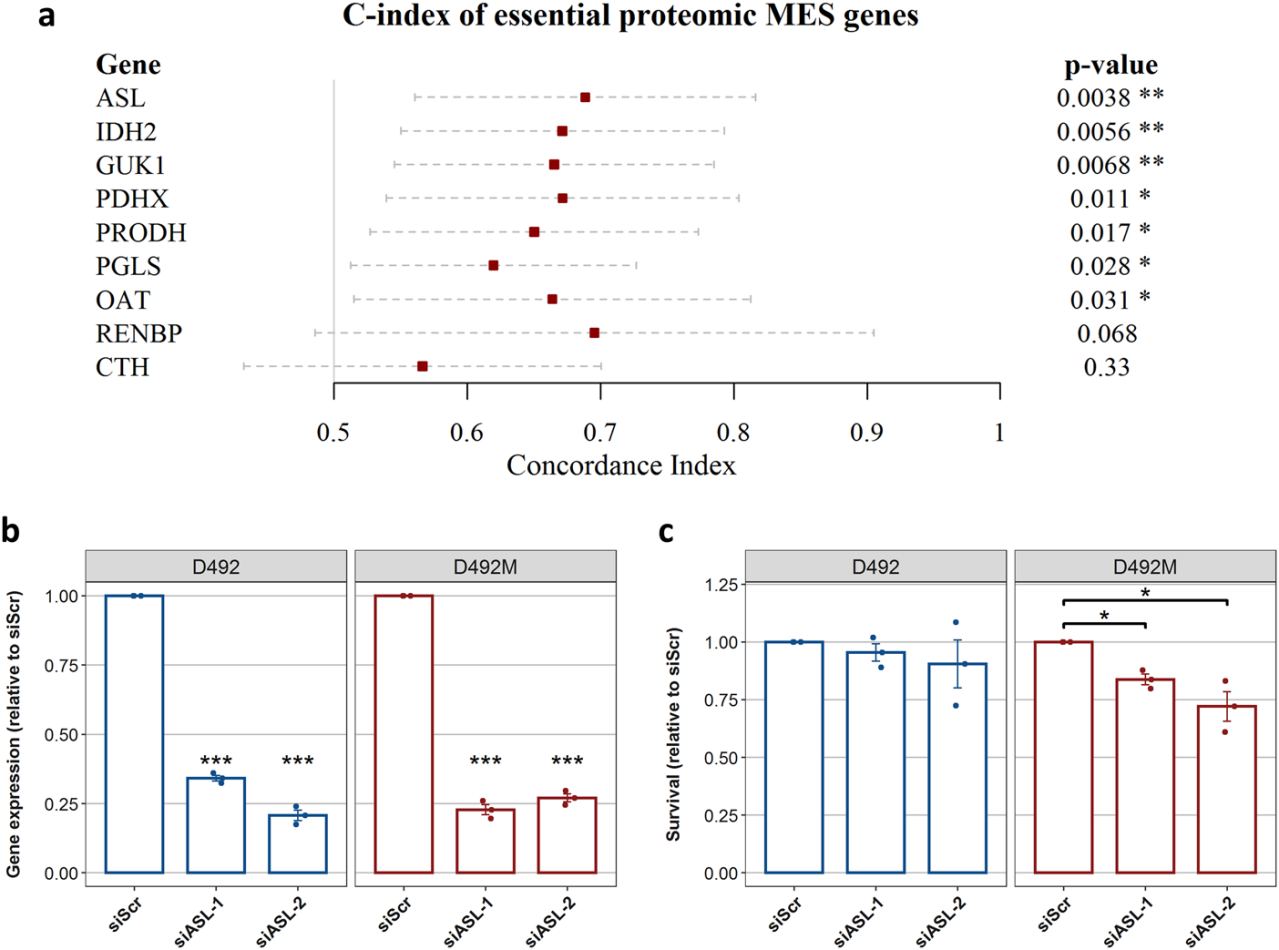
Selection and knockdown of MES-essential genes. **a** Concordance index (C-index) of proteomic MES essential genes for overall survival prediction of patients with claudin-low breast tumors. Lines represent 95% confidence intervals and *p* values are from the calculation of the C-index using Noether’s method^79^. Genes are plotted in descending order based on the *p* values. **b** Expression levels of ASL in D492 and D492M after siRNA-mediated knockdown of the gene. Two different siRNA constructs were used for ASL (ASL-1 and ASL-2) (**c**) siRNA-mediated knockdown of ASL and its effects on the 96 h survival of D492 and D492M. Results in (**b**) and (**c**) are shown as mean + SEM from three experiments (shown with dots). Student’s *t*-test was used to estimate significance and *p* values were adjusted using the Benjamini–Hochberg approach.

In order to narrow down the list of genes to verify in vitro, we evaluated the genes‘ relationship to survival of patients with claudin-low breast cancer, which is representative for the mesenchymal-like phenotype of breast cancer that expresses high levels of EMT markers^27^. This we achieved by measuring the concordance index (C-index) for the genes, which is a metric for predictive ability of survival models based on gene expression levels^28^. *ASL* had the highest C-index (and lowest associated *p* value) among the genes (Fig. 5a) and was chosen for in vitro survival analysis.

After lowering *ASL* expression by 75% using small interfering RNAs (siRNA), the survival of D492M cells was significantly diminished whereas the survival of D492 cells was not altered (Fig. 5b and c). The same effect was observed when *GUK1*, the gene with third-lowest *p* value, was silenced in the cells using siRNA (Supplementary figure 3). Importantly, no effect on survival was observed after silencing the gene coding for the neighbor reaction of *ASL*, argininosuccinate synthase (ASS1) (Supplementary figure 3).

### iBreast2886-dependent analysis of breast cancer proteome reveals subtype-specific vulnerabilities

Building on the verification of the gene essentiality predictions, we next validated the ability of iBreast2886 to identify growth vulnerabilities in breast cancer that could potentially be exploited for diagnostic or therapeutic purposes. To achieve this, we used proteomic data from breast tumors^29^ as constraints for the model. Again, we chose proteomic data (instead of available transcriptomic data) based on our preliminary constraint-based analysis with D492 and D492M data which showed its relatively higher accuracy in capturing intracellular flux phenotypes compared to transcriptomic data.

We hypothesized that we would identify subtype-specific metabolic vulnerabilities of breast cancer, i.e. genes specifically essential for estrogen-receptor positive (ER-positive) and ER-negative tumors. The status of the estrogen receptor has repeatedly been shown to be a significant prognostic marker, where patients with ER-negative tumors generally have shorter survival times. After creating 55 patient-specific proteomic GSMMs, we performed gene essentiality analysis on all models. Subsequently, the ER-negative and ER-positive patient GSMMs were tested for over-representation of essential genes using empirical *p* value calculations (see Materials and Methods).

A single essential gene was identified for ER-negative patients: Argininosuccinate Lyase (*ASL)* (empirical *p* value = 0.0419). In order to confirm these results, we acquired the metadata for the patients through the Gene Expression Omnibus (GEO) and performed survival analyses using survival in months as time and cancer-related death as event.

Univariate and multivariable Cox proportional hazard models were constructed using age and *ASL* separately for patients with ER-positive and ER-negative tumors. The Cox analyses (shown in Table 1) revealed that although *ASL* protein levels were a predictor of death in the univariate models of patients with both ER-positive and ER-negative tumors (HR = 1.16 and 1,12; *p* = 0.067 and 0.049, respectively), the inclusion of age in the multivariable models attenuated the effects of *ASL* in the ER-positive patients (HR = 1.08, *p* = 0.44) but not in ER-negative patients (HR = 1.12, *p* = 0.062). Thus, after adjusting for confounding effector age we observed that *ASL* was a marginally significant predictor of cancer-related death of only ER-negative patients, which confirms the results from the gene essentiality analysis using breast cancer proteomic data and iBreast2886.

**Table 1:** Univariate and multivariable Cox proportional hazard models suggest proteomic levels of ASL are significantly associated with survival of ER-negative breast cancer patients.

		Univariate			Multivariable		
ER-status	Variables	HR	95% CI	*p* value	HR	95% CI	*p* value
Positive	ASL	1.16	0.99–1.35	0.067	1.08	0.89-1.31	0.44
	Age	1.07	1.03–1.12	0.0022	1.06	1.02-1.11	0.0086
Negative	ASL	1.12	1.00–1.25	0.049	1.12	0.99-1.25	0.062
	Age	0.99	0.94–1.05	0.77	1.00	0.95-1.05	0.93

The models were created using age (in years) and ASL protein levels. The event used in the survival analysis was cancer-related death.

There were no genes significantly enriched for ER-positive patients, likely due to the heterogeneity of this breast cancer subtype which might require further stratification based on the status of the progesterone and HER2 receptors or genes within the PAM50 panel^30^.

## Discussion

High-throughput molecular screening can serve to focus experimental efforts on understanding the functional consequences of molecular variation. Here we have used genome-scale metabolic network modeling to reverse this classification and prioritization strategy. Rather than using high-throughput clinical data as the basis for network analysis of generic metabolic models, we used GSMMs constrained with data from cells in culture whose metabolic phenotypes resemble breast gland development to describe the metabolic landscape of breast epithelium and identify changes in metabolism associated with breast cancer.

A comparison of the different omics data from the breast epithelial cells D492 and their mesenchymal isogenic cell line D492M revealed a low correlation of the mRNA and protein levels, compatible with literature reports on the correlation of these data types^22,31–33^. There was an even lower correlation of the two different mRNA quantification methods microarray and RNA sequencing (Fig. 1) which is in accordance with the previous studies^34^.

The correlation of transcriptomic and proteomic data can be compartment-specific due to the different spatiotemporal nature of the molecules^35^. Accordingly, after constraining iBreast2886 with all the different omics data, we found that the differences in fluxes between the proteomic- and RNA-seq-constrained models were indeed highly compartment-specific (Supplementary table 1). Three compartments (cytosol, mitochondria, and the ER) had a significant correlation of *ρ* > 0.37, with the ER having the highest value of 0.54. A plausible explanation is that mRNA is synthesized in the nucleus, but is subsequently exported to the rough ER where protein translation takes place. Therefore, the ER displaying high correlation of reaction activity based on proteomic and transcriptomic data is unsurprising^36,37^.

Multiple factors influence the consistency of proteomics and transcriptomics data, not only technical ones like experimental discrepancies and different data-producing platforms, but also biological factors like gene regulation, post-translational modification, different rates of synthesis, and availability of resources^35^. Our findings support that these are different based on the type of cellular compartment and show that the accuracy of GSMMs flux predictions from extracellular uptake and secretion measurements is dependent upon the transcriptomic and proteomic profiles of the cellular compartment of interest.

The compartment-dependent correlation results highlight that care must be taken in the interpretation of metabolic phenotypes from high-throughput data as these may fail to accurately represent the most fundamental parts of energy metabolism. Indeed, it was apparent that the predicted relative activity between EPI and MES was highly data type-dependent (Fig. 2) with the proteomic-constrained GSMMs predicting flux phenotypes most similar to measured pathway activity using a 1-^13^C-glutamine tracer (Fig. 3). Further analysis of the proteomic GSMMs was successful in proposing valid changes to D492 metabolism following EMT. The EMT-linked reaction list was enriched particularly with reactions taking part in cholesterol and squalene metabolism (Fig. 4a). As a confirmation of these predictions, we found that the cholesterol-inhibiting drug lovastatin had a significantly stronger effect on the survival of D492 than D492M cells (Fig. 4d). Cholesterol has previously been shown to be a promoter of EMT^38^ and the cholesterol-inhibiting drug statin has been shown to inhibit cancer invasion and metastasis^39–41^. Importantly, the differences in cholesterol metabolism of D492 and D492M were not captured by a general KEGG pathway enrichment analysis of the significantly different proteins in the cell lines (Supplementary figure 4), suggesting the presence of emergent properties of the iBreast2886 network that are biologically relevant and cannot be elucidated using a generic differential expression analysis.

Similarly, the gene essentiality analysis for the proteomics-constrained MES model suggested that argininosuccinate lyase (*ASL*) would be essential for D492M which was confirmed by in vitro siRNA knockdown experiments (Fig. 5). Upon knockdown of the gene, there was a 22.1% reduction in survival of D492M cells on average in contrast to only 7% in D492 cells. This level of survival reduction in D492M is comparable to results from validations in previous studies using similar methodology proposing metabolic targets, where 10-80% reduction in survival have been observed upon in vitro knockdown of the main metabolic target genes^14,42^. A manual investigation of the GSMM flux profiles revealed three likely reasons for the essentiality of *ASL*: 1) compromised proline synthesis via *OAT* accompanied by 2) decreased fumarate production for the TCA cycle and 3) decreased OAA to aspartate conversion that compromises aminotransferase activity and therefore anaplerotic fueling of the TCA cycle. In addition to ASL, we identified six other significant targets from the gene essentiality analysis, most of which have been associated with poor cancer survival^6,43–45^. For example, increased expression of the *IDH2* gene has been shown to be overexpressed in endometrial, prostate, testicular, and advanced colon cancer^46–48^, and we have recently demonstrated that IDH2 indeed fuels reductive glutaminolysis and fatty acid synthesis in the D492 and D492M cells in an accompanying study^49^.

Several studies have carried out metabolic network analysis on a compendium of clinical transcriptomic or proteomic data to extract and prioritize metabolic features of importance^14,15,50–52^. Using our breast metabolic GSMM iBreast2886, we analysed publically available breast tumor proteomic data from 55 breast cancer patients and identified *ASL* as a metabolic vulnerability of the aggressive ER-negative breast cancer.

Thus, we found that *ASL* was both essential for D492M cell growth and related to the worse prognosis of ER-negative breast cancer patients. The enzyme coded by *ASL*, argininosuccinate lyase, produces fumarate and arginine from the breakdown of argininosuccinate. Arginine is a non-essential proteogenic amino acid involved in nitrogen detoxification and the generation of nitric oxide (NO) which is important for invasion and metastasis in various cancer types^6,53^. Downregulation of *ASL* has been shown to inhibit the growth of breast cancer tumors in vitro and in vivo^54^. Rabinovich et al.^55^ reported that downregulation of *ASS1*, an enzyme directly upstream of *ASL*, increased pyrimidine synthesis and cancer cell proliferation but did not see the same connection with *ASL*. The different effect of siRNA knockdown of *ASL* and *ASS1* on D492 and D492M survival reported here support a mutually exclusive relationship of *ASL* and *ASS1* as only *ASL* and not *ASS1* was found to be essential for growth of D492M (Fig. 5c and Supplementary figure 3). This however does not explain the observed differences in the context of linear pathway flux within iBreast2886. A possible explanation is a secondary function of *ASL*, as it has been shown to influence cyclin A2 levels by direct binding in hepatocellular carcinoma, independent of its enzymatic activity within the *ASS1-ASL* node that also promoted anchorage-independent growth^56^. Intercellular exchange of argininosuccinate between *ASL*- and *ASS1*-deficient cells, as demonstrated by Davidson et al.^57^, furthermore indicates that the two enzymes need not be co-regulated within a single-cell type. This type of tissue-level metabolic crosstalk would not be captured by our single-cell metabolic reconstruction iBreast2886. Nevertheless, the components of the *ASS1-ASL* node, citrulline, and fumarate, have been reported to be significantly lower and higher, respectively, in ER-negative breast cancer compared to ER-positive which supports altered activity within the ASS1-ASL metabolic node^58^. The findings additionally support more studies that have shown that metabolic vulnerabilities of breast cancer lie within arginine metabolism^54,59,60^.

Taken together, the study demonstrates that the metabolism of EMT captured within iBreast2886 is practical for data integration and analysis and that proposed phenotypes are in agreement both with prior investigations of EMT/metastasis and ER-negative breast cancer metabolism. The iBreast2886 reconstruction is first and foremost a metabolic model descriptive of the steady-state metabolic phenotypes that the D492 EMT cell model can achieve based upon the integration of mRNA transcription, protein translation, and metabolite uptake and secretion rates. The integrated analysis of multiple iBreast2886 GSMMs constrained with separate data types collectively yielded more accurate predictions than each individual GSMM, as shown here with the EMT-related changes in cholesterol metabolism and *ASL* essentiality.

Limitations of iBreast2886 include lack of actual measurements of fatty acid oxidation and cholesterol uptake/secretion rates which might further increase predictive accuracy of iBreast2886. Genes involved in the oxidation of fatty acids are known to correlate with reduced cancer patient survival^14,61,62^ and the relationship of cholesterol to EMT and metastasis has been discussed here above^38–41^. The robustness and plasticity of breast tissue metabolism are also more complex than is captured by iBreast2886, which is solely based upon one EMT cell culture model and media constraints that may not accurately reflect the breast tissue microenvironment^63^ and lack flux extremities that may arise from kinetic regulation. Steps towards further understanding of EMT metabolism could be performed by expanding iBreast2886 to account for additional cell lines alongside focused studies aimed at addressing metabolic gaps and network inconsistencies whose presence was demonstrated in this study using isotope tracer analysis. In this way, biochemically accurate descriptions of EMT metabolism in breast tissue to aid in translational cancer research may be pushed forward.

## Materials and methods

### Cell culture

D492 and D492M were cultured in a serum-free H14 medium at 37*°*C and 5% CO2 as previously described^19^. H14 is a fully defined medium comprised of DMEM/F12 base with 250 ng/ml insulin, 10 µg/ml transferrin, 10 ng/ml EGF, 2.6 ng/ml sodium selenite, 10^*-*10^ M estradiol, 1.4 µM hydrocortisone, 7.1 ng/ml prolactin, 100 IU penicillin, 0.1 mg/ml streptomycin and 2 mM glutamine. Medium was changed every 48 h while propagating cells, and experiments were performed within four passages. D492 and D492M cells were kindly provided by the Stem Cell Research Unit, University of Iceland, and were screened for *Mycoplasma* infections monthly using PCR-based tests at the Biomedical Center, University of Iceland.

### Origin of iBreast2886 GSMM for breast metabolism

Genome-scale metabolic model construction and analysis were carried out in MATLAB using the COBRA Toolbox^64^. The genome-scale breast tissue metabolic model from Halldorsson et al.^16^ was used as a base model. Briefly, RNA sequencing data from both the D492 and D492M cell lines^19^ was used to create a metabolic model specific for breast tissue. To achieve this, the human metabolic reconstruction Recon 2 was employed^65^. All genes in the RNA sequencing data with expression values exceeding a fixed cut-off value (1 RPM) in either cell line were identified. To identify the metabolic reactions associated with the list of genes, the Gene-Protein Rules (GPRs) of Recon 2 were used. The FASTCORE model building algorithm^66^ was subsequently applied to build a functional metabolic network from the list of identified reactions. The resulting network, referred to as the iBreast2886 model, was manually curated to ensure no metabolites or pathways were blocked or missing.

### Construction and analysis of cell-type-specific epithelial and mesenchymal GSMMs

The iBreast2886 reconstruction was used to create cell-type-specific models of epithelial D492 and mesenchymal D492M based on microarray, proteomic, and RNA sequencing (RNA-seq) data. The workflow of the model construction is outlined in Supplementary figure 5. Briefly, the genes/proteins from each dataset (along with cell-type-specific uptake and secretion rates of multiple metabolites in the media) were used to constrain iBreast2886 to create two models (EPI for epithelial D492, and MES for mesenchymal D492M). Furthermore, the fourth pair of EPI and MES was added that did not contain any intracellular constraints imposed by omics data, but only the cell-type-specific uptake and secretion rates. This gave rise to four pairs of EPI and MES models, where each EPI model shared the same stoichiometry and uptake/secretion rates but had different intracellular reaction constraints based on the different datasets. The same applied to the MES models. See Supplementary information for details.

### Stable isotope tracing analysis

D492 and D492M cells were cultured until confluent as described above. The medium was then changed to a complete H14 containing 1-^13^C-labeled glutamine (Cambridge Isotope Laboratories, Inc., MA, USA). After 6 h of culturing with the ^13^C-labeled carbon source, cell metabolism was quenched using cold methanol and intracellular metabolites were extracted using ACN extraction^67^. Analyses were performed on a UPLC system as described in Rolfsson et al.^68^. Results were presented as the total contribution (TC) of carbon sources to measured metabolites^69^:

1 $$TC = \frac{{\mathop {\sum }\nolimits_{i = 0}^n i\cdot m_i}}{n}$$

Where *n* is the number of C atoms in the metabolite, *i* represents the isotopologues and *m* is the relative fraction of the isotopologues.

### Comparison of GSMM flux predictions and ^13^C-labeling profiles

Individual flux distributions from constraint-based modeling of GSMMs were used to estimate the relative contribution of extracellular metabolites to intracellular metabolites of interest. This approach is suitable to measure the flow of carbons between metabolites within GSMMs to ultimately quantify the total activity of specific metabolic routes within pathways that can subsequently be directly compared to definitive results from stable isotope tracer analyses. A schematic explaining the metabolic route activity measure is shown in Supplementary figure 6. A single flux distribution represent the flux values of all reactions within a GSMM which is subject to the constraints applied to the model. In order to calculate the relative flux value $v_{rel}$ from metabolite $m_i$ to $m_{i + 1}$ within a pathway of interest, we first identify all consuming reactions of metabolite $m_i$ using the stoichiometric matrix *S*. Then, for a single flux distribution, one can calculate the sum of consuming flux of metabolite $m_i$, and the relative flux that is used to produce only metabolite $m_{i + 1}$, which we will call $v_{rel}$. If *k* is a consuming reaction of a particular metabolite of interest, then the $v_{rel}$ value for *k* is calculated from the raw flux value of *k* divided by the sum of the fluxes of all *K* reactions consuming the same metabolite as *k*. Therefore, the $v_{rel}$ of *k* (or $v_{rel}(k)$) in a single flux vector is calculated as follows:

2 $$v_{rel}(k) = \frac{{v(k)}}{{\mathop {\sum }\nolimits_{i = 1}^K v(i)}}w_{comp}$$

Where $w_{comp}$ is the weight given to the relative flux value based on the relative abundance of the compartment it takes place in, since some reactions take place in more than one compartment. The $v_{rel}(k)$ values for all transport reactions were assumed to be 1. The weights for the compartments were as follows: Cytosol 0.54, mitochondria 0.22, ER 0.12, nucleus 0.06, golgi apparatus 0.03, peroxisomes and lysosomes 0.01, and are representative of their relative volume within cells in general^70^.

Using the relative consumption values for a list of reactions within a single flux vector, it is possible to calculate the metabolic route activity (MRA). To calculate the MRA from metabolite *m* to *m* + *k*, calculate the sum of the log of relative flux values (from Eq. (2)) within that route:

3 $$MRA = \mathop {\sum }\limits_{i = 1}^{k - 1} \log \left( {v_{rel}\left( {m_{i + 1}} \right)} \right)$$

Where the first reaction is the consumption of metabolite $m_i$ to produce metabolite $m_{i + 1}$. The MRA of multiple flux vectors (e.g., within a random sampling matrix) can be calculated to get a distribution of MRA within a specific constrained GSMM.

### Lovastatin assay

D492 and D492M cells were treated with 5, 10, and 100 µM concentration of lovastatin (Tocris Bioscience, Bristol, UK) for 24 h after which both cholesterol abundance and cell numbers were assessed. The cholesterol was measured using Amplex™ Red Cholesterol Assay Kit (Thermo Fisher Scientific, Waltham, MA, USA) according to manufacturers protocol. The cell numbers were assessed using crystal violet staining. Briefly, after 24 h of treatment, the cells were fixed using ice-cold methanol and stained with crystal violet (0.5%). The stain was subsequently released using 10% acetic acid and absorption was measured at 570 nm.

### Scoring of *in silico* gene essentiality candidates

The METABRIC breast cancer clinical dataset^71^ was downloaded from cBioPortal^72,73^. The clinical metadata includes information about the claudin-status of the tumors in the data. Using only patients with tumors classified as *claudin-low* and available survival data (*n* = 199), we performed a survival analysis. In short, patients were split into two groups based on the best-splitting expression level (as identified through R-package *survminer’s* surv_cutpoint() function) of a gene of interest. The prognoses of the groups were then examined by calculating the concordance index (C-index)^28^, which provides an overall measure of predictive accuracy of the genes’ expression level with right-censored survival data.

### Small interfering RNA (siRNA) knockdown experiments

For the knockdown experiments, Silencer Select siRNAs (Thermo) were used (Negative Control No 1 #4390843), *ASL* (s1669 and s1671), and *ASS1* (s1684). Cells were seeded at density of 3000 cells/well in a 96 well plate. Prior to seeding, the 96 well plates were coated with siRNA and Lipofectamine RNAiMAX (Thermo) for 15 min. Final concentration of siRNA in each well, after addition of cells, was 10 nM. Transfected cells were incubated at 37 °C and 5% CO_2_ for 96 h at the end of which cell survival and gene expression were assessed. To measure cell survival, CellTiter Glo Luminescent Cell Viability Assay (Promega, Madison, WI, USA) was used according to instructions of the manufacturer. SpectraMax plate reader was used to measure luminescence at 560 nm. To measure the gene expression, real time quantitative polymerase chain reaction (qPCR) was used.

### Real-time PCR

Total RNA was isolated using TRI-Reagent (Thermo) according to the manufacturer’s instructions. RNA concentration was measured using NanoDrop One (Thermo). 0.4 to 1 ug RNA was reverse transcribed to cDNA using High-Capacity cDNA Reverse Transcription kit (Thermo). Real-time quantitative PCR reactions were carried out using Luna Universal qPCR Master Mix (New England Biolabs, Ipswich, MA, USA) according to manufacturer’s instructions on a BioRad CFX384 Touch™ Real Time System (BioRad Laboratories, Hercules, CA, USA). Gene expression levels were determined with CFX Manager Software (BioRad). Primers were designed using the Primer3 software^74^. Primers spanning exon junctions were chosen to ensure specificity. Differences in relative expression were estimated using the 2^∆∆Ct^ method. The primer sequences used for quantifying the gene expression were: ASL-fwd 5‘-GGAAGCTGTGTTTGAAGTGTCA-3‘, ASL-rev 5‘-CCATGTTCTCTTGGTGAATCTG-3‘, ASS1-fwd 5‘-CAGGAAAGGGGAACGATCAGGT-3‘, ASS1-rev 5‘-CGTGTTGCTTTGCGTACTCCAT-3‘, GUK1-fwd 5‘-CTTCATCGAGCATGCCGAGTTC-3‘, GUK1-rev 5‘-GAACCTGTATGGCACGAGCAAG-3‘, ACTB-fwd 5‘-CTTCCTGGGTGAGTGGAGACTG-3‘ and ACTB-rev 5‘-GAGGGAAATGAGGGCAGGACTT-3‘.

### Analysis of clinical breast cancer data using iBreast2886

Proteomic breast cancer data were acquired from Tang et al.^29^. After removing identifers with missing data in more than 20% of samples, the data were imported into MATLAB for constraint-based modeling.

Patient-specific GSMMs were constructed from iBreast2886, where the reactions were only constrained in a patient model if their associated protein levels were below the 60th percentile in all patients. The same amount of constraint was applied to the selected reactions as for the EPI and MES models (as described above). The median percentage of constrained reactions in the patients was 3.8%. Gene essentiality analysis was carried out using FBA as described above.

Essential genes that were over-represented in the GSMMs of estrogen receptor (ER) negative (n_1 _= 33) and positive patients (n_2 _= 32) were identified by randomly sampling n_1_ and n_2_ patient-specific GSMMs 1000 times from the whole GSMM list. Then, an empirical *p* value ($\hat p$) was calculated for each gene in the ER-negative and ER-positive patient subsets using the formula from North et al.^75^:

4 $$\hat p = \frac{{r + 1}}{{n + 1}}$$

Where $\hat p$ is the empirical *p* value, *n* is the number of resampled sets (1000 in this case) and *r* is the number of times the resampled sets have an equal or greater number of an essential gene compared to the ER-negative or ER-positive patient sets.

Genes with a $\hat p$ < 0.05 were identified and their proteomic levels^29^ were tested as subtype-specific survival predictors using the patient metadata acquired from GEO (GSE37751). The metadata used were cancer-related death and survival in months that were acquired using the R-package GEOquery^76^. To assess the effects of genes and confounding variables on patient survival, Cox-proportional hazard models were employed using the R-package survival^77^.

### Statistical analysis

For comparison of two groups, a two-sided Student’s t-test was employed unless when the data did not follow a normal distribution, when the non-parametric Mann–Whitney *U*-test was used. When more than a single treatment was compared in the cell lines, the treatments were all compared to the negative control using two-sided Student’s *t*-test and subsequently, the *p* values were adjusted for multiple comparisons using the Benjamini–Hochberg approach. For the comparison of two distributions (e.g., in the metabolic route activity measurements), a Kolmogorov–Smirnov test was used. Presented data were from at least three independent experiments (represented by dots) and were summarized using mean + standard error. The asterisks in each figure represent the *p* values (*<0.05, **<0.01, ***<0.001, ****<0.0001, ns = not significant). Statistical methods used for GSMM analysis of breast cancer patients are described in the Analysis of clinical breast cancer data using iBreast2886 section. All statistical analysis was carried out using the R programming language^78^.

### Reporting summary

Further information on research design is available in the Nature Research Reporting Summary linked to this article.

## Supplementary information

Supplementary Information

Supplementary Data 1

Reporting Summary

## Data Availability

The datasets generated during and/or analysed during the current study are available from the corresponding author on reasonable request. The breast cancer proteomic data that were analysed during the current study are available in Tang et al.^29^ and the metadata were acquired from the Gene Expression Omnibus (GEO), accession no. GSE37751.

## Code availability

All code used in this study for constraint-based modeling and analysis are accessible at https://github.com/siggitrausti/EMT_metabolic_modeling.
