# Supplementary material for: Argininosuccinate lyase is a metabolic vulnerability in breast development and cancer
Source: NPJ Syst Biol Appl. 2021 Sep 17;7:36. doi: 10.1038/s41540-021-00195-5 (PMC8448827; doi:10.1038/s41540-021-00195-5)
Supplement: Supplementary file 1 — Supplementary Information [file 41540_2021_195_MOESM1_ESM.docx]

**Supplementary information**

**1. Materials and methods**

**1.1. Construction of cell-type specific epithelial and mesenchymal GSMMs**

To model the metabolism of D492 and D492M, intracellular reactions within iBreast2886 breast model was constrained using cell-type specific omics data and extracellular uptake and secretion rates were bounded by measured uptake and secretion rates of the two cell lines (**Supplementary figure 4**). In this study, the accuracy of three different omics data in predicting the metabolic phenotypes of D492 and D492M were compared. These omics data were 1) microarray, 2) proteomic and 3) RNA sequencing data. Using each of these datasets, the breast tissue GSMM iBreast2886 was used to create two GSMMs, EPI and MES, representative for the metabolism of D492 and D492M, respectively. The first step in the analysis of all the datasets was the identification of genes and proteins that are downregulated in D492 and D492M to use for intracellular reaction constraining.

*1.1.1. Identification of genes/proteins for cell-type specific GSMM creation*

Microarray expression data for D492 and D492M^1^ were obtained and analysed as described in Halldorsson *et al*.^2^. Briefly, based on a sensitivity analysis, the genes with log-fold relative difference of > +5 in D492M compared to D492 were used to constrain reactions in EPI and genes with log-fold relative difference of < -5 used to constrain reactions in MES. Using the Gene-Protein-Rules (GPRs) in iBreast2886, the reactions associated with the genes were identified. A total of 544 reactions were constrained for both EPI and MES (9.4% of all iBreast2886 reactions).

Proteomic data for the D492 and D492M cells were acquired from Wang *et al.* (Wang et al., submitted). The dataset contains proteomic measurements of both cell lines in triplicate. The levels of all proteins were log_2_-transformed and proteins with more than one measurement missing in both cell lines were omitted. The proteomic levels in D492 and D492M were then compared using Student‘s t-test. The p values were adjusted for multiple comparisons using Storey‘s FDR-controlling approach^3^. The UniProt identifiers of the proteins were converted to Entrez idenfiers using the R/Bioconductor package *biomaRt*^4,5^ to make them consistent with the gene identifiers in the iBreast2886 reconstruction. To facilitate a fair comparison between the proteomic-constrained and microarray constrained GSMMs, a similar number of constrained reactions was desired. A threshold for FDR-adjusted p-values of < 0.05 resulted in a total of 444 constrained reactions for EPI and MES (7.1% of all iBreast2886 reactions).

RNA sequencing (RNA-seq) data for D492 and D492M were obtained from Halldorsson *et al*.^2^. This dataset contains HUGO identifiers and FDR-adjusted p-values. The HUGO identifiers were converted to Entrez identifiers using *biomaRt*. Again, we sought to constrain a similar amount of reactions in the RNA-seq GSMMs as done in the microarray and proteomic-constrained GSMMs. A threshold of FDR-adjusted p-values of < 0.05 resulted in a total of 513 constrained reactions (8.9% of all iBreast2886 reactions).

The lists of reactions to constrain based on the three different omics data are shown in **Supplementary file 1**. After the reactions to constrain were obtained from the omics data, the next step was to apply these constraints to the generic breast reconstruction iBreast2886.

*1.1.2 Creation of cell-type specific GSMM creation*

A lower bound was imposed on the biomass function in iBreast2886. The value chosen as the lower bound was half of the maximal biomass production rate obtained from a flux balance analysis (FBA)^6^ of the model optimizing biomass production. This means that all of the feasible flux vectors in the solution space of iBreast2886 produce some amount of biomass. Prior to adding further constraints based on the different omics data, random sampling of the solution space^7^ of iBreast2886 was performed to estimate the distribution of feasible flux values for each reaction. This was achieved using the gpSampler function in the COBRA Toolbox^8^, an Artificial Centering Hit-and-Run (ACHR) algorithm which generates flux vectors that satisfy the constraints and the steady-state assumption of the model. For the iBreast2886, 5800 flux vectors were randomly sampled over 32 hours.

Next, the iBreast2886 model was constrained using the omics data of different origin to generate the EPI and MES GSMMs. The genes or proteins significantly upregulated in D492M were linked to reactions using the iBreast2886 GPRs and used to constrain the flux boundaries in the associated reactions to generate the EPI models. For creation of the MES models, the significantly upregulated genes/proteins in D492 were used. Instead of simulating a complete inhibition of reaction activity, the reactions were constrained to the 30th percentile of the their flux values based on the random sampling results from the iBreast2886 GSMM by adjusting the upper and lower bounds of allowable flux values. The upper and lower bounds for the rest of the reactions were set as the maximum and minimum flux values, respectively, from the random sampling of iBreast2886.

After the intracellular reactions had been constrained to create EPI and MES GSMMs using each dataset of different origin, the extracellular uptake and secretion rates in the models were adjusted according to exometabolomic data measured for both D492 and D492M^2^. These targeted exometabolomic data contain measurements of the media concentrations of 39 metabolites over 48 hours of cell culture of both D492 and D492M. The time-dependent concentration values, along with the growth rate of the cells and their dry weight, were used to calculate the cell type-specific uptake and secretion rates of the metabolites as shown in the following equation:

| $v_{k}=\frac{V\left( \left[ M_{k} \right]_{f}-\left[ M_{k} \right]_{i} \right)}{A}$ | (1) |
| --- | --- |

|  |  |
| --- | --- |

Where $v_{k}$ is the exchange rate for metabolite k, $\left[ M_{k} \right]_{48}$ and $\left[ M_{k} \right]_{0}$ are the concentrations of metabolite *k* in the culture media after 48 and 0 hours, respectively, and *A* is the area under the growth curve.

At this stage, we have EPI and MES GSMMs constrained with three different types of omics data and exometabolomic measurements. These are the microarray GSMMs, proteomic GSMMs and RNA-seq GSMMs. As a control, we added another set of EPI and MES GSMMs where no intracellular reactions were constrained with any omics data, but the uptake and secretion rates were constrained on a cell type-specific level using the exometabolomic data. These are referred to as media GSMMs.

A stoichiometric matrix, *S*, contains information about the connection of metabolites and reactions in GSMMs. All EPI and MES models share the same stoichiometry *S* but have different constraints on reaction bounds defined by different abundances of metabolic genes/proteins and different uptake/secretion rates defined by the exometabolome. The workflow of the model construction is outlined in **Supplementary figure 4.**

**1.2 Constraint-based modeling and analysis of context-specific GSMMs**

If the bounds of reactions in a GSMM are too stringent, then there may not be any feasible flux vectors, i.e. flux vectors that satisfy the constraints of the model. If the cell type-specific EPI or MES models were infeasible once the intracellular and extracellular constraints were applied, the bounds of the reactions were minimally adjusted so that feasibility was achieved. This was achieved using an algorithm previously described^2^ which is formulated as the following minimization problem:

| minimize **∑***_j_***_∈_***_R_*_r_ **(** *p*_j_ + *n*_j_ ) |  | (2) |
| --- | --- | --- |
| **Sv** = **0** |  | (3) |
| *l*_j_ **-** *n*_j_ **≤** *v*_j_ **≤** *u*_j_ **+** *p*_j_ | *j* **∈** *R*_r_ | (4) |

The objective is to minimize the total adjustment of model reactions (2) by adjusting the relaxation of their upper ($p_{j}$) and lower ($n_{j}$) bounds while satisyfing the steady state assumption (3). The set of reactions to be relaxed is denoted by $R_{r}$ (4). The algorithm was implemented in MATLAB using the CVX software for convex programming^9^.

Random sampling of the EPI and MES models was performed as described in the previous section for iBreast2886. This was done after placing a lower bound on the biomass function for each model corresponding to 50% of their maximum growth rate based on flux balance analysis (FBA) where the biomass function was optimised. The median of the randomly sampled flux values for each reaction was identified for all cell type-specific GSMMs. In this manner it is possible to generate a single representative flux vector for the EPI and MES models which does not reflect the maximisation of biomass production (like what would be achieved through FBA optimizing biomass production), an unrealistic objective of healthy human cells.

To identify the minimal adjustments needed to divert an EPI flux phenotype towards a MES flux phenotype, representative of the EMT-linked metabolic alterations that occur, a minimization of metabolic adjustment (MOMA)-based algorithm was used. This algorithm finds the minimal alterations in reaction bounds needed for an EPI model to display a specific MES flux vector. The optimization problem is formalised in the following manner:

$$minimize \left\| v-v_{MES} \right\|_{1}$$

|  |  |
| --- | --- |

Where $v$ are the decision variables, $v_{MES}$ is the median flux vector of MES and $\left\| . \right\|_{1}$represents the Manhattan norm (1-norm). The algorithm returns a list of reactions in EPI whose bounds need relaxation in order to obtain a flux phenotype that resembles that of MES. In our study, we used this list of reactions to perform a hypergeometric test to identify whether the altered reactions were enriched with any subsystems (*e.g.* the metabolic pathway families with specific functional roles) within iBreast2886. This was achieved using COBRA Toolbox‘s fluxEnrichmentAnalysis (FEA) function.

Gene essentiality analysis for all GSMMs was performed using the singleGeneDeletion function within the COBRA Toolbox. This function identifies genes whose inactivation directly affects the predicted growth rate of the models. The genes whose *in silico* deletion caused a complete inibition of biomass production were labeled as essential.

**2. Results**
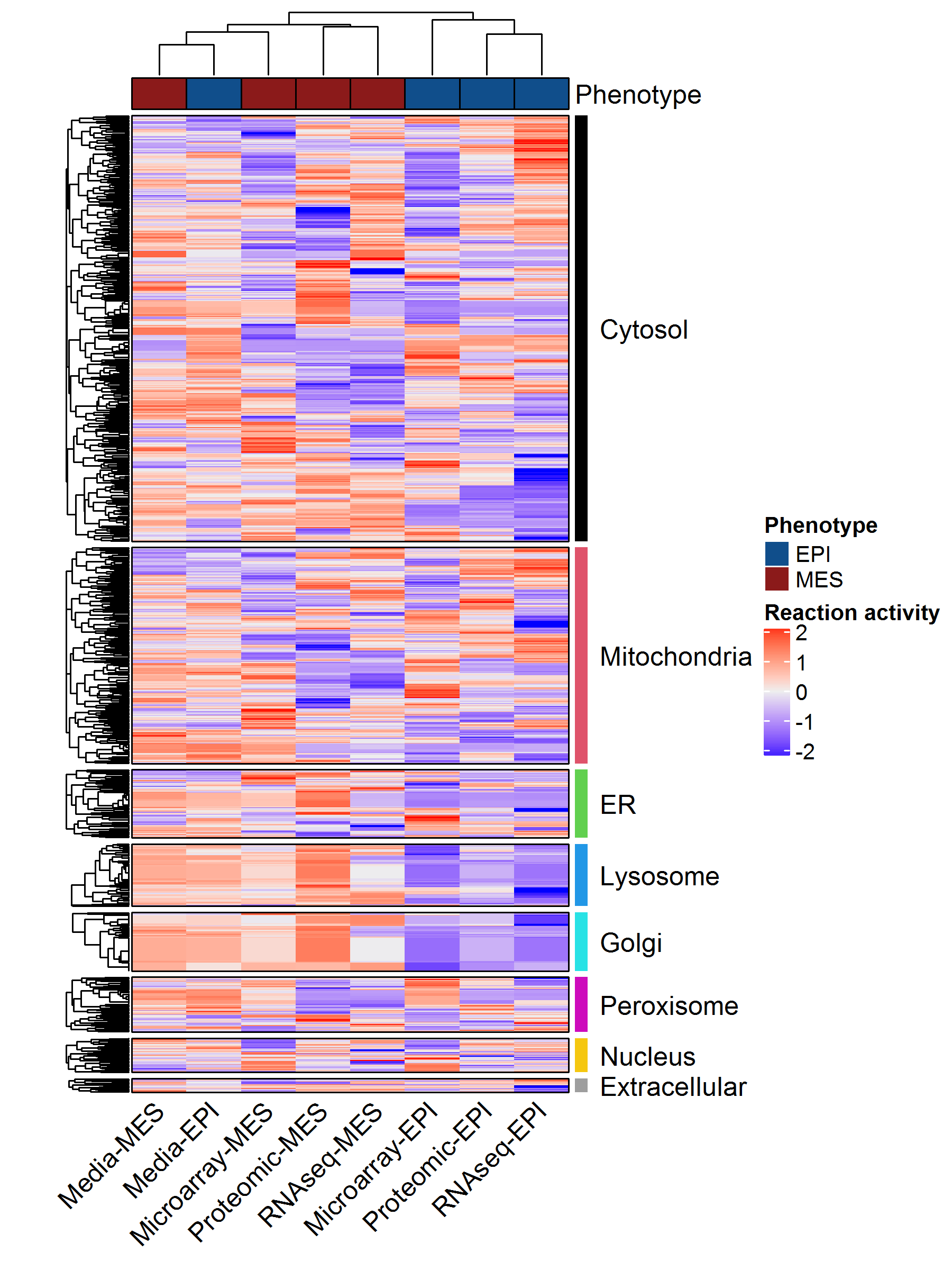


**Supplementary figure 1**. **Heatmap of flux profiles of all GSMMs constrained with different data types.** Hierarchical clustering reveals higher similarity between the EPI and MES phenotypes of differently constrained GSMMs then EPI and MES GSMMs constrained with the same data type. The flux profiles are the median values for each model reaction after random sampling of the GSMMs solution space. Reaction activity is scaled and reactions are shown as members of different subcellular locations (row annotations). The represent the different GSMMs, where blue are EPI GSMMs and red are MES GSMMs The flux vectors are clustered using hierarchical clustering where complete linkage was used as a distance measure.

**Supplementary table 1**. **Spearman correlation coefficient for relative differences in EPI and MES fluxes between proteomic- and RNA-seq-constrained GSMMs.** The log-fold relative differences in reaction activity between EPI and MES was used as a measure for each reaction in both omics-constrained GSMM pairs. The correlation coefficients (and p-values) between the two omics types were calculated from these measures. The adjusted p-value represents the Bonferroni-adjusted Spearman correlation p-values.

| Compartment | Spearman correlation | p-value | Adjusted p-value |
| --- | --- | --- | --- |
| Cytosol | 0.39 | 0 | 0 |
| Mitochondria | 0.37 | 2.2E-12 | 1.7E-11 |
| Endoplasmic reticulum | 0.54 | 1.1E-09 | 8.6E-09 |
| Lysosome | -0.1 | 0.31 | 1 |
| Golgi apparatus | -0.29 | 0.0047 | 0.038 |
| Peroxisome | -0.021 | 0.85 | 1 |
| Nucleus | 0.29 | 0.029 | 0.23 |
| Extracellular | -0.0051 | 0.98 | 1 |


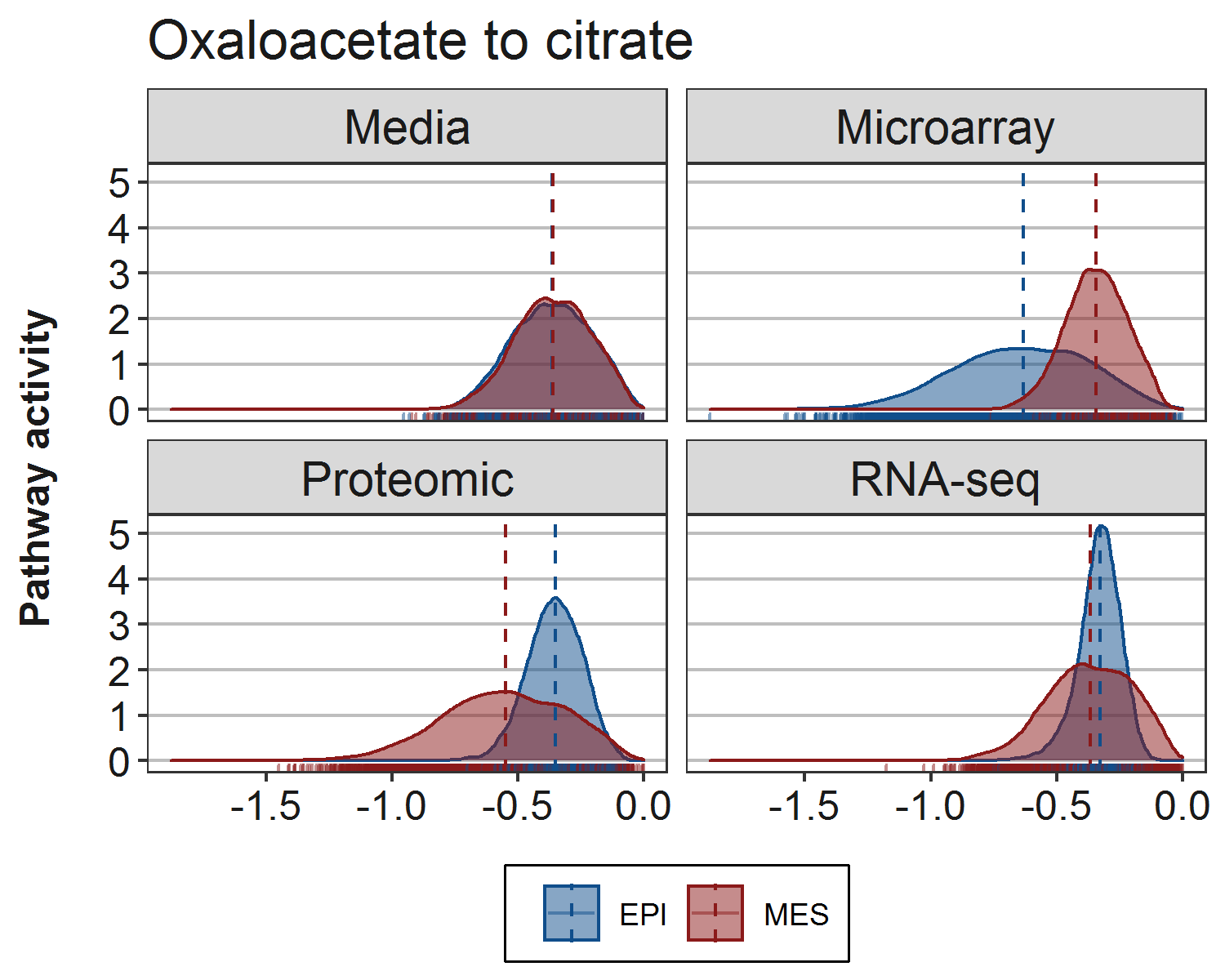


**Supplementary figure 2. Density plots of the calculated metabolic route activitiy (MRA) of the condensation of oxaloacetate and acetyl-CoA to generate citrate**. Results are from the total random sampling matrix (n = 5800 flux vectors) for all GSMMs. The blue distributions represent the MRA within the epithelial GSMMs whereas red represents MRA within the mesenchymal GSMMs. The dashed line represents the median MRA value. Higher (i.e. more positive) values represent more active routes. All distributions were significantly different (p < 0.05) based on a Kolmogorov-Smirnov test.


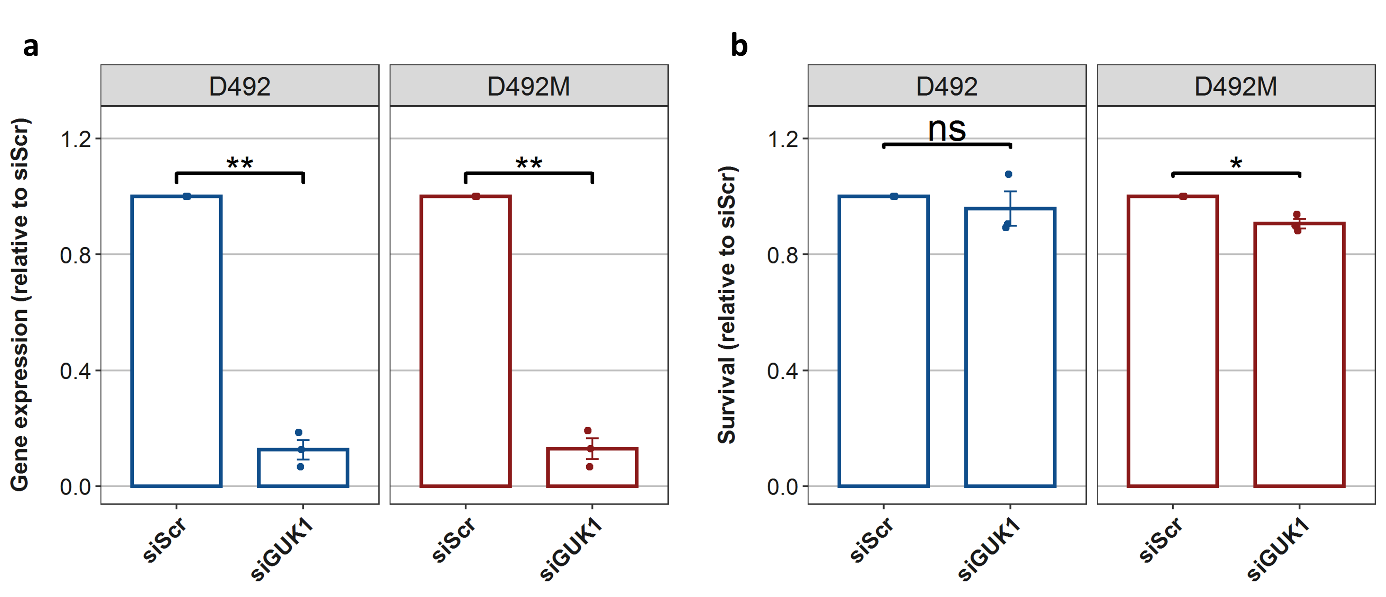


**Supplementary figure 3. Knockdown of GUK1 but not ASS1 inhibits survival of D492M.** A) Expression levels of GUK1 and ASS1 in D492 and D492M after 96 hours of siRNA-mediated knockdown of the genes.) C) siRNA-mediated knockdown of GUK1 and ASS1 and their effects on the 96 hour survival of D492 and D492M. Results in A and B are shown as mean + SEM from three experiments (shown with dots). Student‘s t-test was used to estimate significance and p-values were adjusted using the Benjamini-Hochberg approach.


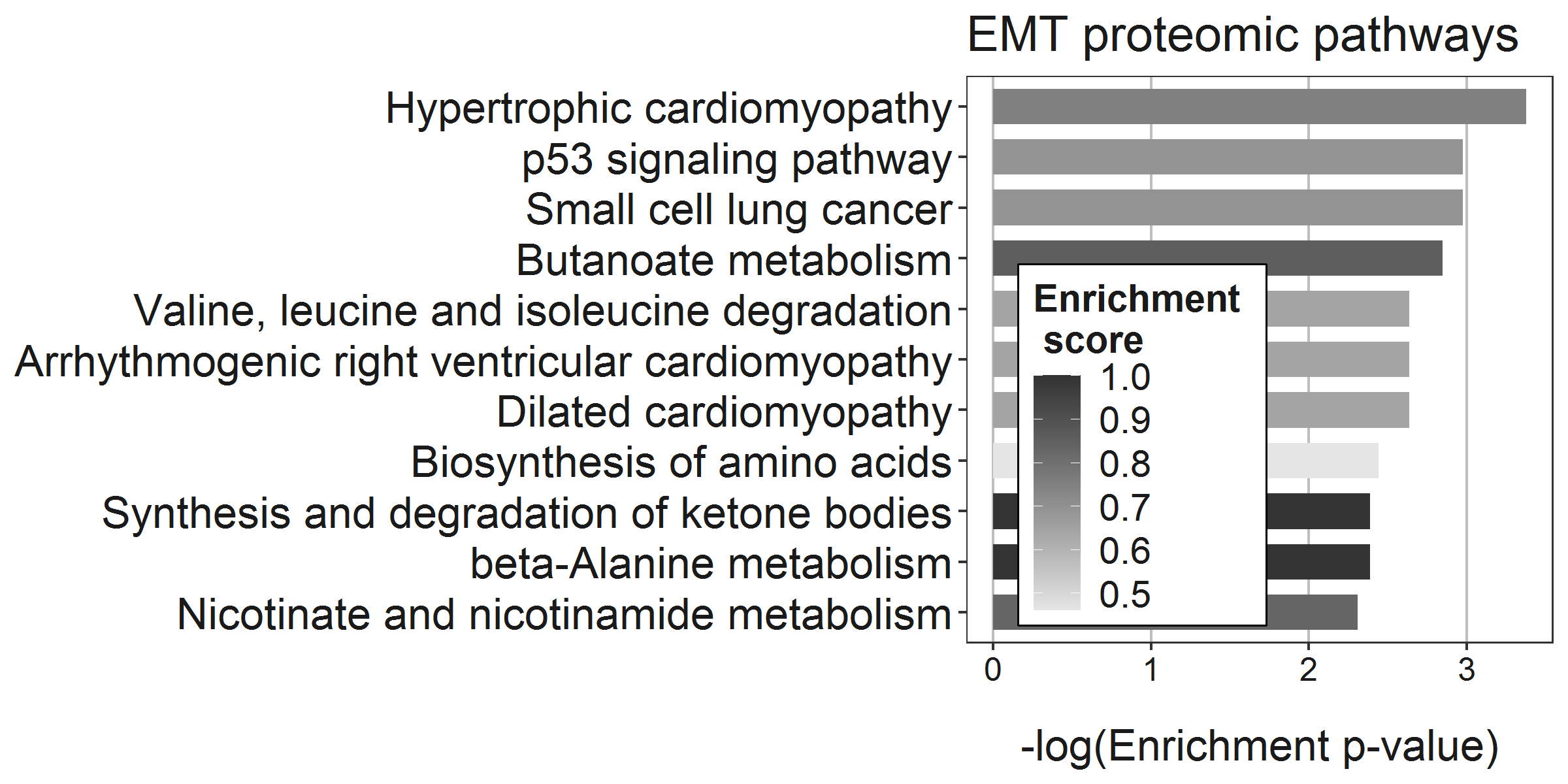


**Supplementary figure 4.** **Enriched KEGG pathways within the differently expressed proteins in D492 and D492M.** After identifying the proteins with FDR-adjusted p-value < 0.01 (n = 588), a KEGG enrichment analysis was performed using a one-sided hypergeometric test. This was performed using the R-package limma, where the background used was the total set of proteins with at least 2 replicates present in either cell line on average (N = 2307). The pathways shown are the ones with p < 0.01. The scale (Enrichment score) represents the fraction of pathway identifiers within the significantly different proteins.


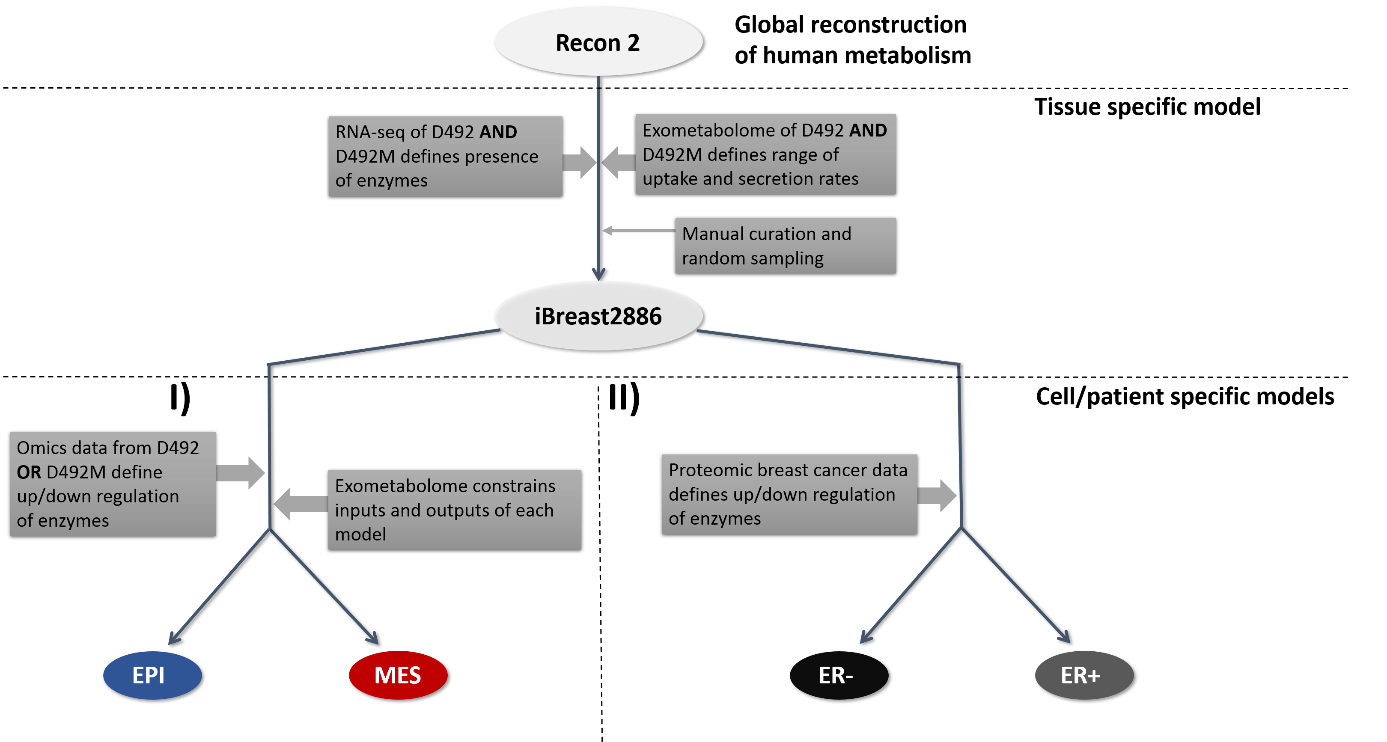


**Supplementary figure 5. Workflow for generating cell- and patient- specific genome-scale metabolic networks. The** networks are descriptive of the metabolism of I) D492 and D492M and II) Breast cancer data from the Tang et al.^10^. The generation and the manual curation of iBreast2886 was previously done by Halldorsson et al.^2^.

.


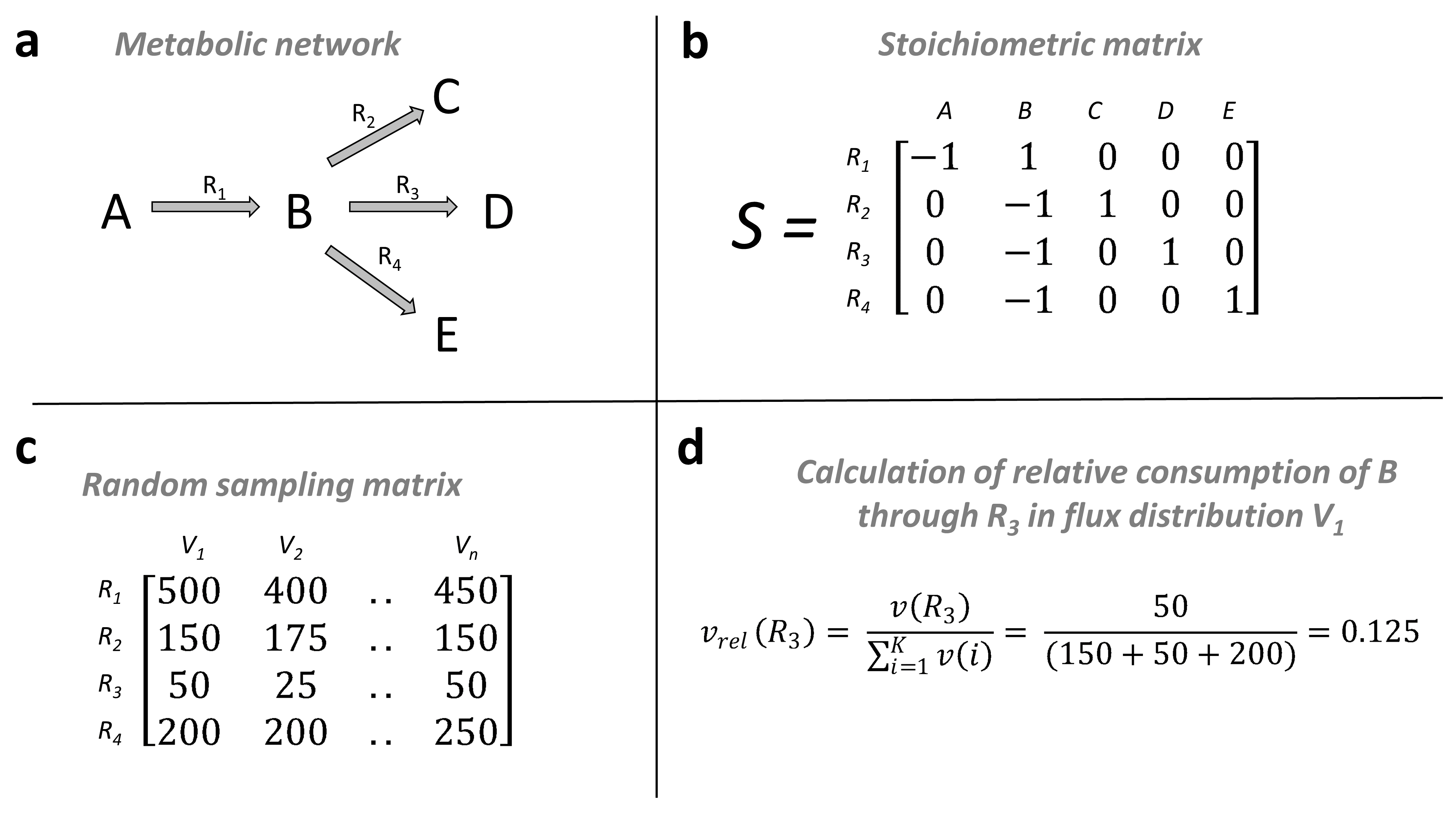


**Supplementary figure 6. Explanation of the metabolic route activity calculations of the GSMMs.** a) A simple, metabolic network comprised of metabolites A-E and reactions R_1_-R_4_. b) A stoichiometric matrix for the metabolic network in A, showing reactions in rows and metabolites in columns. The coefficients represent the abundance of metabolites taking part in each reaction and whether they are consumed or produced. In this example, there are three reactions in which metabolite B is consumed, i.e. in reactions R_2_, R_3_ and R_4_ as these have a -1 for this metabolite. c) An example of a random sampling matrix of the metabolic network in a), subject to the steady state assumption and some additional constraints of the model‘s reaction (not shown here). Briefly, there are n flux distributions in the sampling matrix (each column). d) Calculation of the relative consumption of B through the R_3_ reaction in flux distribution V_1_ in the random sampling matrix in c). In this case, the relative contribution of R_3_ to the consumption of B is 0.125.

**References**

1. Sigurdsson, V. *et al.* Endothelial Induced EMT in Breast Epithelial Cells with Stem Cell Properties. *PLoS One* **6**, e23833 (2011).

2. Halldorsson, S. *et al.* Metabolic re-wiring of isogenic breast epithelial cell lines following epithelial to mesenchymal transition. *Cancer Lett* **396**, 117–129 (2017).

3. D. Storey, J. *A Direct Approach to False Discovery Rates*. vol. 64 (2002).

4. Durinck, S. *et al.* BioMart and Bioconductor: a powerful link between biological databases and microarray data analysis. *Bioinformatics* **21**, 3439–3440 (2005).

5. Durinck, S., Spellman, P. T., Birney, E. & Huber, W. Mapping identifiers for the integration of genomic datasets with the R/Bioconductor package biomaRt. *Nat. Protoc.* **4**, 1184–1191 (2009).

6. Orth, J. D., Thiele, I. & Palsson, B. O. What is flux balance analysis? *Nature Biotechnology* vol. 28 245–248 (2010).

7. Schellenberger, J. & Palsson, B. O. Use of randomized sampling for analysis of metabolic networks. *J Biol Chem* **284**, 5457–5461 (2009).

8. Heirendt, L. *et al.* Creation and analysis of biochemical constraint-based models using the COBRA Toolbox v.3.0. *Nat. Protoc.* **14**, 639–702 (2019).

9. CVX Research, I. {CVX}: Matlab Software for Disciplined Convex Programming, version 2.0. (2012).

10. Tang, W. *et al.* Integrated proteotranscriptomics of breast cancer reveals globally increased protein-mRNA concordance associated with subtypes and survival. *Genome Med.* **10**, 94 (2018).
